# Quantum System for Generating Random Phase-Manipulated Emissions with a Controllable Electromagnetic Center

**DOI:** 10.3390/s26082329

**Published:** 2026-04-09

**Authors:** Nikolay Litchkov, Momchil Kurtev, Anton Mladenov

**Affiliations:** 1 Institute of Robotics at Bulgarian Academy of Sciences, 1113 Sofia, Bulgaria; litchkov0611@gmail.com; 2 Advanced Flight Technologies Association, 1680 Sofia, Bulgaria; 3 Institute for Nuclear Researches and Nuclear Energy at Bulgarian Academy of Sciences, 1784 Sofia, Bulgaria; anton.mladenov@inrne.bas.bg

**Keywords:** quantum system, phase-manipulated emissions, synchronization of emitted signals, controllable electromagnetic center

## Abstract

**Highlights:**

**What are the main findings?**

- Technical approaches are proposed for using quantum communication technology related to the creation and distribution of quantum keys in a two-channel system for generating phase-manipulated electromagnetic emissions controlled by a practically non-repeatable random code.
- The possibilities for using synchronized signals in both channels of the system have been analyzed, creating a controllable electromagnetic center of their total radiation, which reduces the accuracy of determining the location of the system using radio direction-finding methods.

**What are the implications of the main findings?**

- The quantum system for generating random phase-manipulated emissions with a controllable electromagnetic center can be used to construct bistatic and multistatic radars, communication systems, and other electromagnetic systems with practically infinite (i.e., for the operating time of the system) unique codes for random phase manipulations. The proposed approach can also be used for systems using random frequency-manipulated signals, as well as for those in which the manipulation periods change randomly.
- The proposed approaches can also be used to construct multi-position systems with controllable electromagnetic centers, which significantly increases their concealment, as well as those capable of creating controllable spatial zones of intense or weak total electromagnetic fields for selective electromagnetic interference with radio equipment located in these zones.

**Abstract:**

This paper presents a quantum system designed to generate random, phase-manipulated emissions. A key feature of the proposed system is its ability to create a controllable electromagnetic center. To achieve this, the architecture utilizes two synchronized sources positioned at distinct spatial locations. A method is introduced where Quantum-generated keys are used to form a random sequence in real time to control digital phase manipulators. A block diagram of a quantum system for generating random phase-manipulated emissions with a controllable electromagnetic center has been developed that enables control of the main operating frequency, the length of the additionally generated random sequences controlling the modulations, the frequencies and phases of the emissions, the period and start of phase manipulations, as well as the power of the signals emitted by each of the channels. This way ensures uniformity or a controllable difference in the signals emitted by the two sources of the system upon their arrival at a predetermined point in space. A laboratory prototype of the quantum system has been developed, and tests have been conducted to confirm the feasibility of the proposed method and block diagram. The proposed research refers to a case of phase manipulation of transmitted signals with a preset clock frequency. The theoretical and technical solutions presented in the material can also be used to create systems with randomly frequency-manipulated signals, as well as systems in which the manipulation periods change randomly, determined by random quantum keys generated in real time.

## 1. Introduction

Random phase modulation methods are well known and are used in many radar, communication, and other systems [1,2,3,4,5,6,7,8,9,10,11,12,13]. Random phase-manipulated radiation is obtained by randomly or pseudo-randomly varying the phase of the carrier signal. In practice, this phase change is most often achieved by generating a pseudo-random binary or M-ary sequence (PN sequence). Based on this digital sequence or a set of its symbols (e.g., in QPSK, X-PSK, etc.), the phase change is controlled by a phase manipulator or a phase synthesizer (DDS). The pseudo-random digital sequence is stored in the form of chips, which are elementary time intervals in the pseudo-random sequence (PN) during which the code has a constant value (phase, sign, or level). Each chip has a specific length (chip duration) and changes at a given frequency (chip rate). The code in the random phase-manipulated transmission system is repeated after the end of its period, which is determined by the length of the stored code and the duration of the individual chip [14,15].

In general, many of the systems for creating phase-manipulated emissions operate continuously for days, weeks, or even months. Therefore, the pseudo-random nature of the keys used makes it possible to detect them due to their repetition. In addition, it is possible to determine the location of the transmitting components using radio direction-finding systems that employ various methods, mainly based on determining their bearing. For countermeasures against radio direction-finding systems, radio transmitting devices can use methods such as rapid retuning of operating frequencies, changing their modulation, and even periodic shutdown. Due to the passive nature of radio direction-finding systems, their presence is difficult to detect, but even then, these methods are either not particularly effective or lead to the radio emission system ceasing to function. One of the effective methods of counteracting radio direction-finding measures is to create synchronous interference emitted by two or more modules of the system, which are located at different points in space in order to emit radio signals with a fictitious electromagnetic center [16,17]. This system consists of a block for forming the emitted radio signals, connected to the input of an initial amplification block having several outputs. They are connected to the inputs of a unified cable connection system, the outputs of which are connected to the inputs of the amplification units to the required output level, whose outputs are connected to the inputs for receiving the emitted signals of the antenna systems.

A disadvantage of this system is the possible disruption of synchronization as a result of changes in the signal propagation time in the two branches of the unified cable connection when the temperature and the carrier frequency of the signals change. In addition, the fictitious electromagnetic center is formed only in the direction that is equidistant from the two transmitting modules and cannot be controlled. A common disadvantage is also the limited volume and pseudo-randomness of the signals controlling phase manipulation, which is undesirable for systems designed to operate continuously for a long period of time, creating conditions for multiple code repetition.

Based on the above, it can be argued that finding a solution to two separate problems is of certain theoretical and practical interest. The first is to find a way to create a system with a controllable electromagnetic center of the total emitted signal, and this center can be spatially displaced relative to the location of the actual emitting modules. The second problem is to provide phase manipulation based on a random digital code of practically infinite length, i.e., a code that does not repeat itself regardless of the duration of the system’s operation. In addition, it is also interesting to investigate the possibilities for ensuring uniformity or a controllable difference in the signals emitted by the two sources of the system upon their arrival at a predetermined point in space. This creates controllable spatial zones of intense or weak total electromagnetic fields for selective electromagnetic impact on radio equipment located in these zones.

The main objective of this paper is to propose an approach for generating synchronized random phase-manipulated emissions based on quantum communication systems. It is worth noting that this approach, in and of itself, does not affect the mathematical model of the emitted signals themselves, which is identical to that of any similar type of signal, regardless of how they were generated. At the same time, the present paper does not examine the spatial distribution of the electromagnetic field generated by the proposed quantum-communication-based phase-manipulated and synchronized transmitters, which is a separate and extremely important issue. It requires an in-depth separate study, combined with numerical modeling and additional experiments, in which the stability of synchronization between the two transmitters, the accuracy of their positioning, the influence of the frequencies used, phase manipulation periods, distances between the transmitters, etc., are taken into account.

## 2. Materials and Methods

The methods used in this study to solve the two problems involve analyzing existing materials and technologies and selecting a combination of them that is feasible and enables the formation of random phase-manipulated emissions with a controllable electromagnetic center without repeating the random code used.

To solve the first main problem, it is possible to use the approach of creating uncontrollable radiation with a fictitious electromagnetic center [16,17]. The main idea behind this approach is to form practically identical radiation from at least two synchronized emitters of random phase-manipulated signals located at different points in space. In this case, the total electromagnetic radiation will have an electromagnetic center that, in general, will not coincide with that of each individual emitter. Therefore, such a system could be implemented by using synchronized carrier frequency generators, synchronized by phase switching clocks, modulators connected to phase manipulation control blocks, output amplifiers, and antenna systems. It is assumed that the emissions are spatially isotropic, i.e., that non-directional antennas are used, as in the case of directional antennas, the considerations are valid only for the area in which their patterns overlap spatially. The fictitious electromagnetic center created will be oriented towards a given point in space (let us mark it conditionally with $T_{0}$), assuming that the observer located at this point will determine the center of the system’s radiation as being between the locations of the two transmitting antennas. The main idea behind this approach is to generate practically identical emissions from at least two synchronized generators. To ensure synchronization of the emissions from the two channels of the system, it is necessary to periodically reset the initial phases of the carrier frequency generators simultaneously, which is feasible using modern means.

To control the created common electromagnetic center, it is necessary to control the phase shift of the emissions from the individual emitters and the moments for sending commands from the phase manipulation control blocks. On this basis the phases are switched, so that, taking into account the differences in the distances traveled, the signals at the desired point in space $T_{0}$ are in phase. For the same reason, it is necessary to provide for the control of the amplifications of the output amplifiers so that the energy of the signals at point $T_{0}$ is the same. These capabilities are also possible for practical implementation with modern radio-electronic components.

If the system needs to operate in different frequency ranges, the carrier frequency generators must be controllable and the output amplifiers must be broadband. Obviously, in order to perform the listed functions, the system must have a control unit that operates on command from an operator, based on a pre-set program or algorithm, through built-in artificial intelligence with clearly defined goals, or by a combined method. It is advisable for the control unit’s commands to be transformed into user commands and data, stored and transmitted to other components of the system through separate information blocks.

On this basis, it can be assumed that the main and minimally necessary components of the system providing a controllable electromagnetic center are Control blocks, Information blocks, Controllable carrier frequency generators, Phase manipulation control blocks, Phase modulators, Broadband controllable output amplifiers, and Antenna systems.

To solve the second problem related to ensuring phase manipulation based on a random digital code of practically infinite length, it is possible to use a cryptographic seed-based random number generator (CSPRNG) and periodic reseeding is a possible solution. However, this requires a high degree of synchronization between the two channels. Therefore, it is appropriate to use quantum communication technology related to the creation and distribution of quantum keys in a two-channel system for generating phase-manipulated electromagnetic emissions controlled by a practically non-repeating random code. Quantum key distribution (or quantum communication) is a revolutionary technology for distributing a symmetric secret key using photons or very short laser pulses transmitted through optical fibers or free space. For this reason, quantum key distribution (QKD) is finding increasingly widespread application, primarily for communications security. The basic idea behind quantum key distribution (QKD) is that two communicating parties, traditionally referred to as Alice (A) and Bob (B), can generate a random key at a distance through a specific procedure called a QKD protocol. In this way, security and protection against eavesdropping is guaranteed by the properties of the quantum system, such as the uncertainty principle and the fact that quantum states cannot be copied [18,19,20,21,22].

There is a wide variety of QKD protocols, classified according to some of their characteristics. The main classification distinguishes between Prepare-and-measure protocols (based on the procedure for measuring unknown states in QM) and protocols based on quantum entanglement (using the properties of entangled states) [23,24,25,26,27]. The areas in which the use of quantum systems for communication with quantum encryption is making real progress are constantly expanding [28,29,30,31,32,33], with specific QKD protocols being classified either as discrete variables (DV), which use single-photon detectors and sources of low intensity light, or as continuous variables (CV), which use coherent detectors and sources.

**Figure 1:**
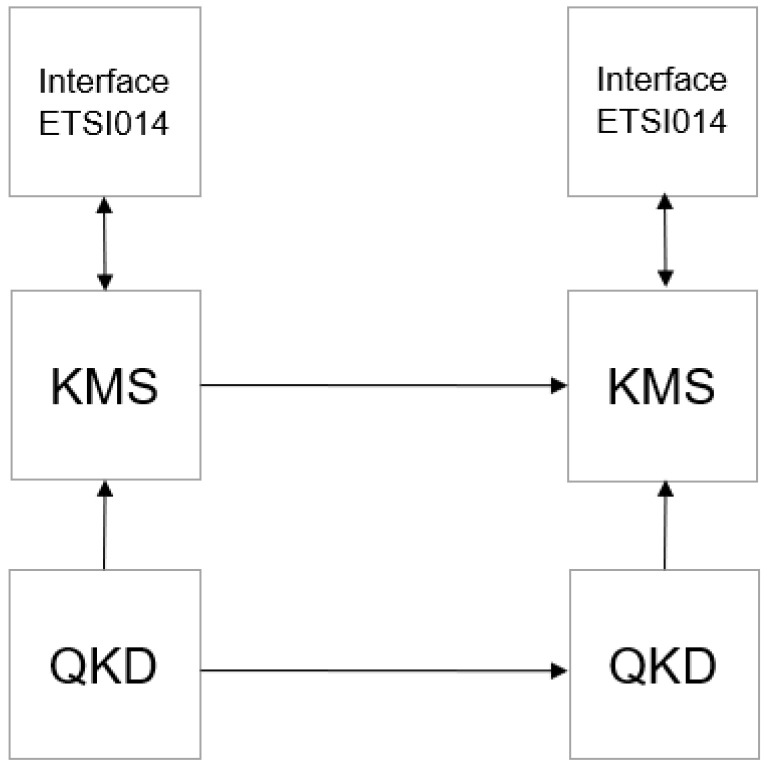
Block diagram of a quantum communication system.

Regardless of its specific type, in general, a quantum communication system includes quantum platforms generating random keys (Quantum Key Distribution—QKD), optical fibers and cables for data transmission, and a key management system (KMS), with its general block diagram shown in Figure 1.

The abbreviations used in Figure 1 are as follows:

- QKD—Quantum Key Distribution;
- KMS—Key Management System;
- Interface ETSI014—interface for communication with KMS;

The quantum keys generated in both parts of the system are absolutely identical and, depending on the specific technical device used, have different durations and are updated in advance at a predetermined and frequently programmable speed. In general, it is possible that the requirements for the frequency of phase manipulation differ from the frequency of updating the quantum keys, i.e., the number of random chips required to control phase manipulation N differs from that provided by the quantum system. Therefore, it is advisable for phase manipulators to be controlled by pseudo-random number generators (PRNGs) with a controllable number of generated numbers equal to N, whose inputs for setting initial states (seed) are connected to the outputs of the Quantum Key Management Systems (KMS) of the respective channel of the Quantum Key Distributors (QKD). In this way, the System will have the necessary number of random numbers generated based on the random numbers of the quantum keys.

On this basis, it can be assumed that the main and minimally necessary components of the system ensuring the solution of the second problem, related to providing a random digital code of practically infinite length for phase manipulation control, are quantum key distributors and quantum key management systems as elements of a quantum communication module, as well as random digital sequence generators associated with them.

## 3. Results

**Figure 2:**
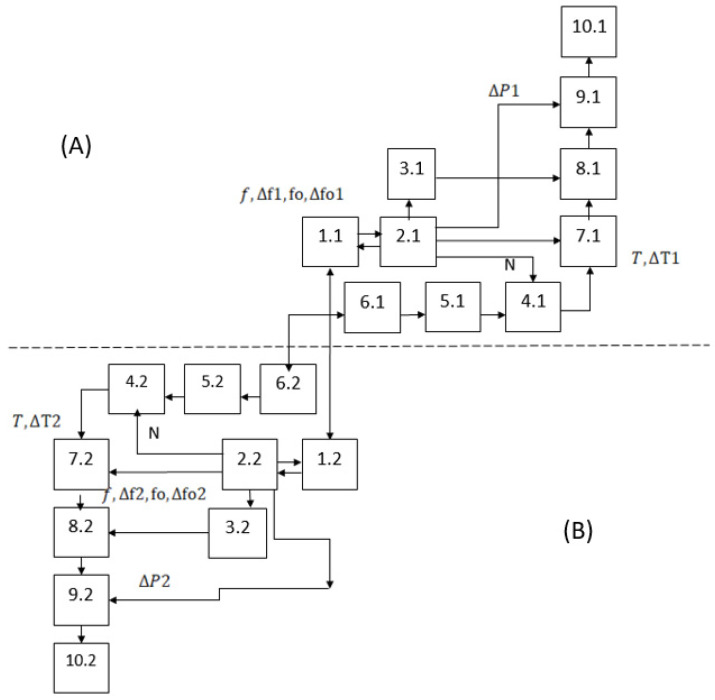
Block diagram of a quantum system for generating random phase-manipulated emissions with a controllable electromagnetic center, (**A**) channel Alice, (**B**) channel Bob.

Taking into account the nature of the selected materials and after analyzing the possibilities for their integration, a block diagram of a quantum system for creating random phase-manipulated emissions with a controllable electromagnetic center, consisting of two similar channels located at different points in space, is shown in Figure 2.

The symbols used in Figure 2 are as follows:

- Control blocks (1.1, 1.2);
- Information blocks (2.1, 2.2);
- Controllable carrier frequency generators (3.1, 3.2);
- Random digital sequence generators (4.1, 4.2);
- Quantum key management systems (5.1, 5.2);
- Quantum key distributors (6.1, 6.2).
- Phase manipulation control blocks (7.1, 7.2);
- Digital modulators (8.1, 8.2);
- Broadband controllable output amplifiers (9.1, 9.2);
- Antenna systems (10.1, 10.2);

The described quantum system for generating random phase-manipulated emissions with a controllable electromagnetic center consists of two similar channels located at different points in space. One of the channels (conditionally named channel A) is the leading one and physically houses the main control unit (1.1). The second channel (conditionally named channel B) is controlled by its own control unit (1.2), connected to the first one via a quantum communication channel.

A characteristic feature of the proposed block diagram is that quantum-generated keys are used to form a random sequence used to control a digital phase manipulator of the transmitted signals, and that it provides the ability to control the main operating frequency (via command f) and changing the length of the generated random number sequences through which phase manipulation is performed (via command N). In addition, there are options for synchronous zeroing of the initial phases and controllable dephasing of the generated signals (via commands $f_{o2},\;{\Delta f}_{o1},\;\Delta f_{o2}$) for introducing dephasing of the generated signals in order to ensure simultaneous and in-phase arrival of the signals emitted by the two channels at a pre-selected point in space.

The control block (1.1) in channel A, upon command from an operator or other source, controls the information block (2.1) synchronously with the control of the analogous control block (1.2) in channel B.

The information blocks (2.1, 2.2) are processor modules which, based on commands from the control blocks and the information for point $T_{0}$, calculate, store, and transmit to users the necessary data for the following controllable parameters: carrier frequency ($f_{0}$), phase deflection (offset) of the generated signal $\Delta f_{1}$, $\Delta f_{2}$, change in the initial phase of the generated signals (${\Delta f}_{o1},\Delta f_{o2}$), period of phase manipulation (T), time delay of the supplied clock pulse ($\Delta T_{1},\Delta T_{2}$) causing phase modulation; time delays of each of the channels ($\Delta T_{1},\Delta T_{2}$) and for the length of the generated sequences of random numbers (N), providing non-repeating commands for phase manipulation.

The implementation of the block diagram shown in Figure 2 ensures the performance of the basic functions of a quantum system for generating random phase-manipulated emissions with a controllable electromagnetic center and a non-repeating random key for controlling phase manipulation.

The controllable carrier frequency generators (3.1, 3.2) are tuned and generate the carrier frequency (f), and to compensate for the phase shifts, a command is periodically received and executed to reset the phase of the emission ($f_{0}$) and to introduce phase dephasing ($\Delta f_{1},\Delta f_{2}$), synchronizing the two channels and ensuring the uniformity of the generated initial oscillations in both channels. Additionally, in order to compensate for the different distances between the antennas of the two channels and the specified point, a change in the initial phase of the generated signals (${\Delta f}_{o1},\Delta f_{o2}$) is introduced. Controllable carrier frequency generators can be implemented based on the ADRF6755 chip for a frequency range from 100 MHz to 2400 MHz, or based on the ADF4355 chip for a frequency range from 51.5625 MHz to 6600 MHz.

Random digital sequence generators (4.1, 4.2) form such a sequence, with each individual digit being used as an output command for phase manipulation of the carrier frequency. They generate and store random sequences of a specified length (N), based on the random digital sequences contained in the quantum keys. Essentially, the random digital sequence generators (4.1, 4.2) are pseudo-random number generators (PRNG) whose inputs for setting the initial states (seed) are connected to the outputs of the quantum key management systems (KMS) of the respective channel of the system (5.1, 5.2), which in turn are connected to the quantum key distributors (QKD) of the respective channel of the system (6.1, 6.2). For the specifically selected phase manipulation frequency, these generators provide the required length of random sequences (N), which does not depend on the length and refresh rate of the quantum keys. The random digital sequence generators can be implemented in software and be part of the overall software product embedded in the processor board.

The phase manipulation control blocks (7.1, 7.2) read the next digit received and stored in the random digital sequence generators (4.1, 4.2) during each clock period with interval T and time shifts $\Delta T_{1}\;$and $\Delta T_{2},$ respectively, and when it changes, they send a control command to change the modulation performed by the digital modulators (8.1, 8.2). The delays $\Delta T_{1}$ and $\Delta T_{2}$ compensate for the different distances between the antennas of the two channels and the specified point at which the signals must be identical or have a controllable difference (with coordinates $x_{0}$; $y_{0}{;z}_{0}$). The phase manipulation control blocks can, for example, be implemented in software and be part of the overall software product embedded in the processor board.

Digital phase modulators (8.1, 8.2) perform phase manipulation of the carrier frequency according to commands from the phase manipulation control blocks (7.1, 7.2). Digital phase manipulators can, for example, be implemented in software and be part of the overall software product embedded in the processor board.

Broadband controllable output amplifiers (9.1, 9.2) amplify the signals to the required degree, apply the specified corrections to the amplification $\Delta P_{1}\;$ and $\Delta P_{2}$ (compensating for the different distances between the antennas of the two channels and the specified point at which the signals must be identical or have a controllable difference—with coordinates $x_{0}$; $y_{0}{;z}_{0}$) and feed them to the antenna systems (10.1, 10.2) with directional or non-directional patterns, which transmit them. Naturally, with directional patterns of the Antenna Systems (10.1, 10.2), they must include a module for synchronous control of their direction so that the transmitted signals are in the same spatial zone. Broadband controllable output amplifiers can be implemented based on the ADH8411S chip for a frequency range from 0.01 GHz to 10.0 GHz, as well as using broadband antennas for different bands in the range from 100 MHz to 6000 MHz developed by the Bulgarian company Technopol Security EOOD.

Synchronization between the two channels using a satellite positioning system (GNSS) is possible [34,35,36,37], but requires a continuous and stable navigation signal. Therefore, for systems designed to operate continuously and in all real conditions, it is recommended that the clocks of the two digital modulators be synchronized simultaneously and that the phases of the two generators be zeroed, either through a dedicated White Rabbit time channel (IEEE 1588v2 + SyncE) with an accuracy of less than 1 ns [38,39], or through an optical time-transfer channel providing a synchronized clock at Alice and Bob. It is also possible to use local PLL generators at Alice and Bob locked to the White Rabbit clock, as well as to use QKD as a logical sync trigger, in which the QKD/KMS channel sends a T_0_ marker (a command of the type “At time T = 123,456,789.000000000, reset the phase”), which is authenticated, contains an accurate timestamp, and is executed locally at Alice and Bob.

By implementing the proposed block diagram, it is possible to create a quantum system for generating random phase-manipulated emissions with a controllable electromagnetic center and a non-repeating random key for controlling phase manipulation. The fictitious electromagnetic center created will be oriented toward a given point in space (let us mark it conditionally with $T_{0}$), assuming that the observer located at this point will determine the center of the system’s radiation as being between the locations of two transmitting antennas.

**Figure 3:**
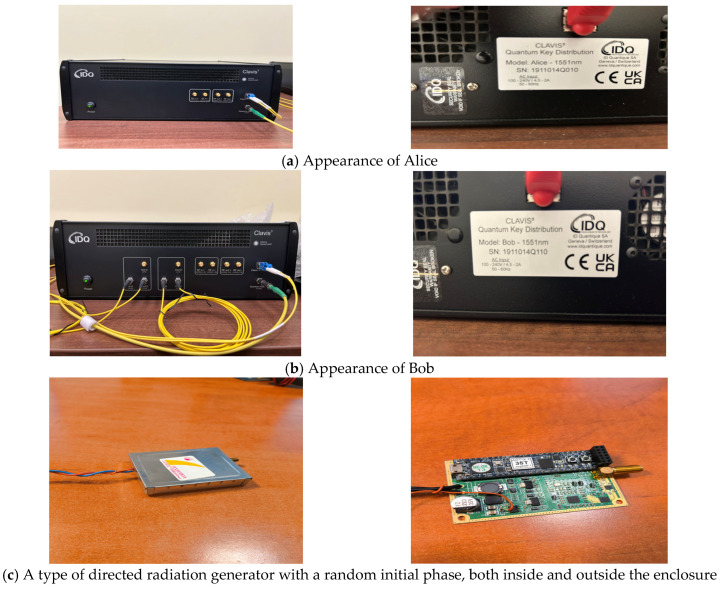
Appearance of Alice (**a**), appearance of Bob (**b**), appearance of the main components of the laboratory model of a quantum system for generating random phase-manipulated emissions with a controllable electromagnetic center (**c**).

Based on the block diagram shown in Figure 2, an algorithm has been developed and a specialized software product has been created to perform the specified operations. A laboratory model of the main devices of a quantum system for generating random phase-manipulated emissions with a controllable electromagnetic center has been implemented. It consists of two communication blocks, conventionally called Alice and Bob (Figure 3a), connected by a Single Mode Fiber Patch Cable using an LC/UPC-LC/UPC Duplex Connector. The other components in the laboratory model are implemented in a processor unit based on FPGA CMOD-A7-35T, placed in a box (Figure 3c). It contains software designed to perform their functions and interfaces for programming, control, and management, as well as a laboratory sample of a power supply module, a controllable carrier frequency generator (3.1, 3.2), a broadband amplifier, and rod-type antenna systems (10.1, 10.2). These modules are connected to the quantum key distribution modules (6.1, 6.2) via the quantum key control modules (5.1, 5.2), with communication with 5.1 and 5.2 being carried out via the standardized etsi014 protocol. Several approaches are possible for the implementation of quantum key management modules: these modules can be separate devices, they can be integrated into other devices, or they can be software-based and integrated into other devices. Depending on the needs and possibilities, a different approach can be used, since conceptually they are the same, as in the laboratory model the quantum key management modules were separate devices.

The laboratory model was set up, calibrated, and successfully launched in a working hall, and then successful real-life tests were conducted with it, confirming the feasibility of implementing the proposed quantum system for creating random phase-manipulated emissions with a controllable electromagnetic center.

## 4. Discussion

### 4.1. General Analysis

It is known that for two coherent non-directional (isotropic) sources of electromagnetic waves located at a distance D, the interference pattern is determined by the difference in the paths of the waves from the two sources (respectively the distance $R_{1}$, which is represented by the line, $T_{0}A$ and distance $R_{2}$ which is represented by line segment, $T_{0}B$) to each point in space. Due to the wave nature of the emitted signals, the interference pattern in coherent emission will represent a sequence of interference waves with maxima (when the two signals are in phase) and minima (when the two signals are out of phase) in the form of double-leaf hyperboloids of revolution with foci coinciding with the location of the two emitting sources [40,41,42]. The interference pattern is a system of surfaces that are symmetrical to the line connecting the two sources and are nested within each other, resulting in hyperbolas when intersected by a plane.

When the phase-manipulated emissions and the moments of phase switching delays are introduced in a manner that ensures in-phase at point $T_{0}$, the type of interference pattern will obviously change so that its maximum passes through point ${\;T}_{0}$. Therefore, by changing the location of point $T_{0}$, a controllable change in the interference pattern of the emitted signals and, accordingly, in the non-equivalent electromagnetic center of radiation of the system is achieved. Obviously, because the differences between the two sides of the triangle are smaller than the third side, and due to the property of the hyperboloid, each maximum, including the one passing through point $T_{0}$, will intersect the line connecting the two emission sources. With precise phasing, the equivalent electromagnetic center of the system will be directed towards the center of the segment connecting the two emitters, and when a known asynchronism is introduced, it can be shifted, but only within its limits.

In general, with two transmitting antennas, it is possible to shift the effective electromagnetic center to any point on the segment between the antennas. When using the same technology to create synchronized and controllable emissions from three or more sources located at different points in space, it is possible to create a fictitious total electromagnetic center at any point in the area between the antennas of these sources.

The determination of the necessary phase shift $(\Delta f_{1},\;\Delta f_{2}$), the time delay of the clock pulse causing phase modulation $(\Delta T_{1},\;\Delta T_{2}$), and the corrections to the output power of each of the channels $(\Delta P_{1},\;\Delta P_{2}$) is based on the difference in the distances between the two emitters and point $T_{0}$, which we can denote as $R_{1}$ and $R_{2}$, respectively. To determine them, let us assume that we use a polar coordinate system with a center located in the middle of the segment connecting the two radiation sources and a zero axis, conditionally accepted as “north,” coinciding with the direction from the second to the first radiation source. Let us denote the coordinates of point $T_{0}$ in this coordinate system as (R, α, γ), where R is the linear distance between the midpoint connecting the two sources and point $T_{0}$, α is the azimuth, and γ is the angle to it.

**Figure 4:**
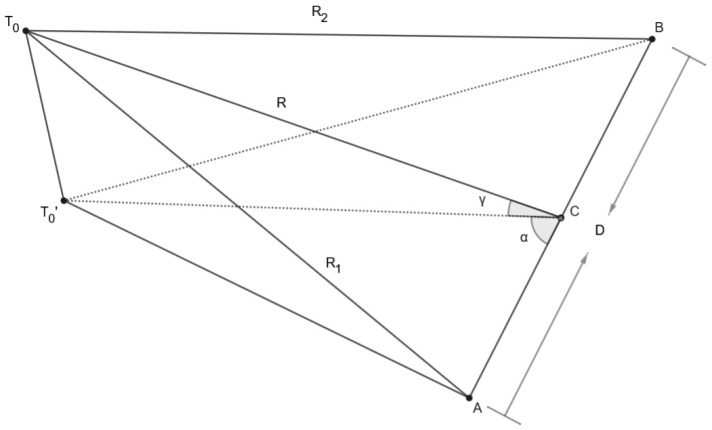
Spatial arrangement of the emitting modules and point.

In this case, the distances ${\;R}_{1}\;$ and $R_{2}$ will be determined based on the ratios shown in Figure 4. 

The symbols in Figure 4 are A and B and are the points where the two emitting branches of the system are located; C is the point in the middle of the segment AB; α—azimuth of point $T_{0}$; γ is the angle at point $T_{0}$,$\;CT_{0}=R$ is the distance between the middle of segment AB and point${\;T}_{0}$; $T_{0}$ is the point for which in-phase or precisely specified phase shift of the signals from the two emitters is required, determined by (R, α, γ). ${T_{0}}^{\prime }$ is the projection of $T_{0}$ onto the horizontal plane in which the two branches of the system are located.

From triangle $\Delta T_{0}C{T_{0}}^{\prime }$, it follows that

(1) $${T_{0}T_{0}}^{\prime }=CT_{0}sin\gamma =Rsin\gamma$$

and that

(2) $${T_{0}}^{\prime }C=Rcos\gamma$$

From triangle $\Delta AC{T_{0}}^{\prime }$ the cosine theorem implies:

(3) $${\left({T_{0}}^{\prime }A\right)}^{2}=\;{\left(AC\right)}^{2}+\;{\left({T_{0}}^{\prime }C\right)}^{2}-2(AC)({T_{0}}^{\prime }C)cos\alpha \;={\left(0.5D\right)}^{2}+{\left(Rcos\gamma \right)}^{2}-DRcos\gamma cos\alpha$$

where D is the distance between the two emitters, i.e., D is the length of the segment AB, and AC is half of its length.

From triangle $\Delta T_{0}{T_{0}}^{\prime }A$, using the Pythagorean theorem it follows that:

$$({T_{0}A)}^{2}=R_{1}^{2}={\left({T_{0}}^{\prime }A\right)}^{2}+{\left(T_{0}{T_{0}}^{\prime }\right)}^{2}\;={\left(0.5D\right)}^{2}+{\left(Rcos\alpha \right)}^{2}-DRcos\gamma cos\alpha +{\left(Rsin\alpha \right)}^{2}$$

From the above equation it follows that:

(4) $$R_{1}^{2}={0.25D}^{2}+R^{2}-DRcos\gamma cos\alpha$$

Similarly, from triangle $\Delta T_{0}{T_{0}}^{\prime }B$, for $\;R_{2}=T_{0}B$ and the law of cosines

(5) $$R_{2}^{2}={0.25D}^{2}+R^{2}+DRcos\gamma cos\alpha$$

Therefore, α<90° when $R_{2}>R_{1}$ and in this case from Equations (4) and (5) it follows that:

$$R_{2}-R_{1}=\Delta R_{21}=\sqrt{{0.25D}^{2}+R^{2}+DRcos\gamma cos\alpha }-\sqrt{{0.25D}^{2}+R^{2}-DRcos\gamma cos\alpha }$$

Similarly, in the same way, when α>90° and $R_{1}>R_{2}$, we obtain

$$R_{1}-R_{2}=\Delta R_{12}=\sqrt{{0.25D}^{2}+R^{2}-DRcos\gamma cos\alpha }-\sqrt{{0.25D}^{2}+R^{2}+DRcos\gamma cos\alpha }$$

In practice, the distant zone of space is usually of interest, i.e., the case where D≪R. Then, from Equation (4), it follows that:

(6) $$R_{1}=R\sqrt{1+{0.25(\frac{D}{R})}^{2}-(D/R)cos\gamma cos\alpha }$$

Considering that the condition D≪R is satisfied, the term inside the square root in Equation (6) can be expanded using a second-order Taylor (binomial) approximation. Retaining terms up to ${\left(\frac{D}{R}\right)}^{2}$ yields a simplified analytical expression for $R_{1}$, which is sufficiently accurate for small baseline-to-range ratios. Such approximations are commonly used in wave propagation and radar geometry analyses when the perturbation parameter is small [43,44].

(7) $$R_{1}\approx R-\left(\frac{D}{2}\right)cos\gamma cos\alpha +\frac{D^{2}}{8R}(1-{\left(cos\gamma cos\alpha \right)}^{2})$$

In the same way described above from Equation (5) we obtain:

(8) $$R_{2}\approx R+\left(\frac{D}{2}\right)cos\gamma cos\alpha +\;\frac{D^{2}}{8R}(1-\;{\left(cos\gamma cos\alpha \right)}^{2})$$

From Equations (7) and (8) it follows that:

(9) $${|R}_{2}-R_{1}|=\Delta R_{21}={|R}_{1}-R_{2}|=\Delta R_{12}\approx Dcos\gamma cos\alpha$$

That is, the modules of the differences between the paths of the electromagnetic oscillations from the two emitters to point $T_{0}$ are equal when the angles α and γ are equal, and when α=90° the difference between these paths is zero. Given the difference in the paths of the two electromagnetic waves for their in-phase arrival at point $T_{0}$, it is necessary to compensate for them by introducing corrections to the emitted signals.

It is known [42,43] that the error Δ  when using Equation (9) is twice the value (sum) of the first non-zero term (in this case, from the third row) when decomposing the expressions from the square root of Equations (7) and (8), which is [42,43]:

(10) $$\Delta \;=\;\frac{D^{3}}{8R^{2}}cos\gamma cos\alpha (1-\;{\left(cos\gamma cos\alpha \right)}^{2})$$

Obviously [42,43], the maximum error value when using Equation (9) Δmax is achieved at the maximum value of the expression $cos\gamma cos\alpha \left(1-{\left(cos\gamma cos\alpha \right)}^{2}\right)$ which, in turn, after equating its first derivative to zero, corresponds to a value determined by the equality $cos\gamma cos\alpha =1/\sqrt{3}$. Therefore, for the case under consideration, for the maximum error when using the approximate Equation (9) and applying the equality cosγcosα = 1/√3, we obtain:

(11) $$\Delta max\;=\;0.0481\frac{D^{3}}{R^{2}}$$

Naturally [44], since the azimuth to point $T_{0}$ is less than 90°, it is necessary to ensure a delay in the phase of the first emitter with $\Delta f_{1}$ defined as $\Delta f_{1}=360.fract(\frac{\Delta R_{21}}{\lambda })$ where λ is the wavelength and $fract(\frac{\Delta R_{21}}{\lambda })$ is the fractional part of the ratio $\Delta R_{21}$ divided by λ. Similarly, at >90°, it is necessary to ensure a phase delay of the second emitter with $\Delta f_{2}$ defined as $\Delta f_{2}=360.fract(\frac{\Delta R_{12}}{\lambda })$.

### 4.2. Sample Calculations

The difference in paths determined by Equation (9) for electromagnetic oscillations from two radiating antennas $\Delta R_{21}$ located in a plane at a distance D from a point with coordinates determined by (R, α, γ) are shown in Table 1 and Table 2, and the requirement for dephasing of the emissions $\Delta f_{1}$ at certain values of the electromagnetic wavelength λ are shown in Table 3.

The data in Table 1 and Table 2 show that the values of $\Delta R_{21}$ depend heavily on the distance between the transmitting antennas and, within relatively wide limits, on the angular position of point $T_{0}$. This justifies the need to use the actual three-dimensional spatial coordinates of the transmitting antennas and point $T_{0}$, rather than approximate ones with a simplified two-coordinate system. For a larger radius of action, in order to determine the difference in the paths of the two emitted oscillations, it is advisable to take into account the refraction of electromagnetic waves and the curvature of the Earth’s surface.

The data in Table 3 show that the values of the necessary phase shifts, in this case the values of $\Delta f_{1}$, depend strongly not only on the angular position of point $T_{0}$, but also on the wavelength. This implies maintaining a stable carrier frequency of the emissions and a high value of coherence of the two channels of the system.

The requirement for dephasing (time shift) of the phase change clocks must compensate for the time it takes for the signals from the two transmitters to reach point $T_{0}$. Naturally, the type of phase modulation must be taken into account so that the start of the modulation cycle of the two sources coincides at point $T_{0}$—for example, in binary modulation (Binary Phase Shift Keying—BPSK) this time is twice as long as the modulation period T, for quadrature (QPSK) it is four times longer than T, and for 8-PSK it is 8 times longer than T. For equal modulation of the signals arriving at point To, it is necessary to ensure the time delay of the modulation of the signal from the transmitter that is closer to point $T_{0}$—i.e., at α less than 90°, the signal from the first transmitter must change only ${\Delta T}_{1}$, and otherwise it must change only by ${\Delta T}_{2}$. The necessary corrections, for example to ${\Delta T}_{1}$ and for BPSK, can be calculated using the expressions:

(12) $$\Delta T_{1}=\frac{\Delta R_{21}}{\;C}-2T\{\frac{\Delta R_{21}}{2TC}\}\mathrm{when}\;\{\frac{\Delta R_{21}}{2TC}\}>1$$

(13) $$\Delta T_{1}=\frac{\Delta R_{21}}{\;C}\mathrm{when}\;\{\frac{\Delta R_{21}}{2TC}\}\leq 1$$

where

$\left\{\frac{\Delta R_{21}}{2TC}\right\}$ is the whole part of the quotient $\frac{\Delta R_{21}}{2TC}$

C—the speed of light in standard atmosphere conditions (299,702,547 m/s).

The data for some possible situations with given coordinates of point $T_{0}$ and phase manipulation period T=100 ns (i.e., with a phase manipulation frequency of 10 MHz) are shown in Table 4.

The data in Table 4 shows that the values of the required time delay of the modulation ${\Delta T}_{1}$ change significantly when the angular coordinates of point $T_{0}$ change. This confirms the need to use an accurate three-coordinate system for the case under consideration, as for longer distances it is advisable to take into account the curvature of the Earth’s surface and the refraction of the emitted signals.

### 4.3. Laboratory Experiment

The laboratory experiment is simplified and is intended to demonstrate the feasibility of the method; based on it, it is possible to develop a more sophisticated experimental setup and conduct experiments on a broader scale. As previously noted, the interference field takes the form of double-leaf hyperboloids of revolution with foci coinciding with the locations of the two emitting sources [40,41,42], which intersects the line segment connecting the two emitters. It varies cyclically in space with a period equal to the wavelength of the emitted electromagnetic waves; therefore, despite the limitations, the experiment provides a clear understanding of it and of the applicability of the proposed method.

To confirm the above, a laboratory experiment was conducted using the components shown in Figure 3 and a quantum communications kit with ID Quantique’s research and development platform (ID Quantique SA, Genève, Switzerland), designed to provide secure and unpredictable randomness for cryptographic applications. This platform is Clavis3 and uses the Coherent One Way (COW) protocol, requiring 4 optical channels: 1 quantum, 2 one-way service, and 1 communication. The Key Management System (KMS) software product (qnet qms 1.1.42) developed by IDQuantique is used to manage the keys, and communication between this KMS and the rest of the equipment is carried out according to the etsi014 protocol.

The laboratory models were implemented as FPGA-based processing modules using the CMOD-A7-35T platform, incorporating the developed software as well as interfaces for programming, control, and system management. The QKD devices were brought into a stable operating state using assistance from IDQuantique’s proprietary software (qnet 1.1.168) for Clavis3, with a quantum channel fiber attenuation for 2 m channel length is of 0.19 dB, an average key generation rate of 1.5 kB/sec, and a Quantum Bit Error Rate (QBER)—which is an indicator of link reliability—is 1.25%; all observed metrics are within the specified limits. An eVHDL implementation of xoroshiro128+ with a seed length of 128 bits was used as the PRNG algorithm. The measurements during the experiment are executed only in a state of stable communication, between Alica and Bob, so that link loss does not affect the results. Non-directional monopole-type antenna systems, RF power supply modules, auxiliary equipment, etc., were employed.

A transmitted signal power of 10 mW, an operating frequency of 149.896229 MHz (corresponding to a wavelength of 2 m), and phase switching cycles of 10 MHz (i.e., every 100 ns), due to the short distance and in order to simplify the laboratory model, no changes were made to the power of the output signals to account for the difference in path lengths. The two branches of the system are located at a distance of 2 m, and the quantum connection between them is achieved via an optical cable. Laboratory tests were conducted for seven characteristic points on a horizontal section connecting the two transmitting antennas, which points are at distances of 0.25 m from each other, starting from the location of the first transmitting antenna. The distances were measured using a Huepar LM100A—100M Laser Distance Meter (Huepar, Zhuhai, China) with a resolution of 1.6 mm, and an NRP-Z81 pulse power measurement sensor connected to a Rohde & Schwarz FSX 20 spectrum analyzer (Rohde & Schwarz, Munich, Germany) capable of measuring pulse sequences with a pulse duration of 100 ns [45,46] is used to measure the power of the total signal. Table 5 shows the data for the required values of the phase shifts $\Delta f_{1},\Delta f_{2},\Delta T_{1}$, and $\Delta T_{2}$, as well as the measured power of the signal emitted by the first antenna ($P_{1}$) and the total measured power (PP) for the seven important measurement points.

The results of the measured power values correspond to the theoretical calculations for the case under consideration and to the relationship between the total power PP, and the powers of each of the emitters ($P_{1}$ and $P_{2}$) at the measurement point, namely [47,48]:

(14) $$PP=P_{1}+P_{2}+2\sqrt{\left(P_{1}P_{2}\right)}cos(\Delta f)$$

where $P_{2}$ is the power of the second emitter at the characteristic point, having mirror values to $P_{1}$, and Δf is the phase shift between the two waves at the characteristic point, which for points up to 1 m is equal to $(\Delta f_{1}$, and then is equal to $\Delta f_{2}$).

To test the possibilities for creating a controllable total electromagnetic field, including the formation of zones with intense or weak total electromagnetic fields, an experiment was conducted to ensure the phasing of the two emissions at a given point and to measure the power of the total electromagnetic field at the seven characteristic points under consideration. Table 6 presents the data obtained, where the phase alignment of the emissions from the two antennas at the respective point is provided in the first column of the table. This phase coherence is ensured by introducing the necessary corrections to $\Delta f_{1}$, $\Delta f_{2}$, $\Delta T_{1}$, and $\Delta T_{2}$, in accordance with the data from Table 5 and the description of the principle of operation of the Quantum system for creating random phase-manipulated emissions with a controllable electromagnetic center, as in the laboratory model there is no control of the output power, which does not change the reliability of the verification of the proposed method.

The results of the measured power values when controlling the emissions so that they are in phase at the corresponding points in the first row of Table 6 correspond to the theoretical calculations, taking into account the fact that in the laboratory model the power of the two emitters is not controlled.

The results presented in Table 5 and Table 6 were obtained by performing 50 consecutive power-up cycles of the laboratory model and measuring the corresponding power levels at each point in space. The results showed the natural fit to a normal error distribution typical for such cases, with the mathematical expectation matching the calculated theoretical values, and the root-mean-square error not exceeding 3.5%.

The laboratory data confirm the possibilities for constructing a quantum system for creating random phase-manipulated emissions with a controllable electromagnetic center and capable of creating zones with intense or weak total electromagnetic fields.

During the tests, stable synchronization between the two channels of the quantum system was recorded, and there were virtually no hardware or software problems.

## 5. Conclusions

The study proposes a block diagram and a general algorithm for the operation of a quantum system for generating random phase-manipulated emissions with a controllable electromagnetic center. This system has two main characteristics: it uses a practically infinite random number sequence to control phase manipulation and controls the equivalent electromagnetic center of the system’s total radiation.

The infinite sequence is proposed to be generated using a quantum-generated random key sequence, i.e., the use of a quantum-generated random sequence with unlimited total length in time. This sequence is used as a seed for a pseudo-random generator (PRNG), which in turn generates the random sequence used to control the phase manipulator of the signal. In this way, by dynamically resetting the pseudorandom generator with quantum-generated keys, a random code sequence with virtually unlimited length is achieved. The implementation of the proposed method depends largely on the distances over which a stable quantum connection can be established, and significant progress has been made in this area [27,32,49].

The controllable total electromagnetic center of the system is ensured by synchronized control of the main parameters of the signals emitted by two identical channels located at different points in space. The control of the necessary corrections of $\Delta f_{1},\Delta f_{2},\Delta T_{1},\Delta T_{2},\Delta P_{1}\;$ and $\Delta P_{2}\;$ can be performed by entering them into the Information Blocks (2.1, 2.2), ensuring uniformity in both phase and anti-phase of the emitted oscillations at the specified point. In this way, it is possible to create and control zones in space where the two emitted electromagnetic oscillations are added together or where they are subtracted.

Most of the research presented in this paper is also applicable to systems with randomly frequency-manipulated signals, as well as to systems in which the manipulation periods change randomly, determined by random quantum keys generated in real time. They can be used in the creation of bistatic and multistatic radars, systems for controllable electromagnetic effects, etc.

In conclusion, we would like to emphasize that the focus of this paper is on the approach to generating synchronized random phase-manipulated emissions based on quantum communications. Future work on this topic involves the creation of an improved experimental prototype, the analysis and selection of one or more suitable metrics for the performance of the proposed system (e.g., spatial contrast of the electromagnetic field, interference fidelity, spatial correlation function, energy focusing efficiency, etc.) and conducting analyses based on experimental results and data from numerical simulations.

## 6. Patents

The article is connected to a relevant patent application, BG/P/2026/114226, at the Patent office of the Republic of Bulgaria.

## Figures and Tables

**Table 1:** Values of $\Delta R_{21}$ for some typical cases where D=250 m.

D	250 m				
γ\ α	10°	25°	45°	60°	75°
10°	242.5	223.1	174.1	123.1	63.7
30°	213.0	196.0	153.0	108.0	56.0
45°	174.2	160.3	125.1	88.4	45.8
60°	123.0	113.0	88.0	63.0	32.0

**Table 2:** Values of $\Delta R_{21}$ for some typical cases where D=1200 m. in degrees.

D	1200 m				
γ\ α	10°	25°	45°	60°	75°
10°	1164.0	1071.0	835.62	590.0	305.8
30°	1023.0	942.0	735	520.0	269.0
45°	835.6	769.0	600.0	424.3	219.6
60°	591.0	544.0	424.0	300.0	155.0

**Table 3:** Required values of $\Delta f_{1}$ in degrees.

D		250 m				
γ\ α		10°	25°	45°	60°	75°
10°	$\Delta f_{1}(0.35\;m)$ $\Delta f_{1}(0.15\;m)$	267	185	144	257	21
		144	192	216	240	288
30°	$\Delta f_{1}(0.35\;m)$ $\Delta f_{1}(0.15\;m)$	68	216	14	103	31
		144	24	216	240	192
45°	$\Delta f_{1}(0.35\;m)$ $\Delta f_{1}(0.15\;m)$	216	339	165	226	278
		24	192	24	168	48
60°	$\Delta f_{1}(0.35\;m)$ $\Delta f_{1}(0.15\;m)$	154	247	195	206	154
		240	96	96	240	240

**Table 4:** $\Delta T_{1}\;$ values in nanoseconds at T=100  ns.

D	250 m				
γ\ α	10°	25°	45°	60°	75°
10°	9.0	144.5	180.9	10.7	12.6
30°	111.4	54.7	110.8	161.2	187.0
45°	181.1	134.8	17.4	95.0	152.7
60°	10.7	178.0	94.9	8.5	107.9

**Table 5:** Values of $\Delta f_{1},\Delta f_{2},\Delta T_{1},\Delta T_{2},P_{1}$ and *PP* for some measurement points.

Measurement Point	0.25 m	0.5 m	0.75 m	1 m	1.25 m	1.5 m	1.75 m
∆f1	270°	180°	90°	0°	0	0	0
∆f2	0	0	0	0	90°	180°	270°
∆T1 ns	5	3	2	0	0	0	0
∆T2 ns	0	0	0	0	1.67	3.34	5.0
P1 mW	13	3	1	1	1	0.04	0.02
PP mW	12.9	1.29	1.9	3.18	1.89	1.29	12.89

**Table 6:** Measured PP values (mW) for some measurement points.

Measurement Point	0.25 m	0.5 m	0.75 m	1 m	1.25 m	1.5 m	1.75 m
0.25 m	17	4	0.2	2	4	4	9
0.5 m	12.9	5.45	1.9	0	1.91	5.46	12.94
0.75 m	9	4	4	2	0	4	17
1 m	12.9	1.29	1.9	3.18	1.89	1.29	12.89

## Data Availability

The data that support the findings of this study are available from the corresponding author upon reasonable request.
